# Options for the diagnosis of high blood pressure in primary care: a systematic review and economic model

**DOI:** 10.1038/s41371-020-0357-x

**Published:** 2020-05-28

**Authors:** Margaret Constanti, Rebecca Boffa, Christopher N. Floyd, Anthony S. Wierzbicki, Richard J. McManus, Mark Glover

**Affiliations:** 1 National Clinical Guidelines Centre, Royal College of Physicians, London, UK; 2 Department of Clinical Pharmacology, King’s College London, London, UK; 3 Department of Metabolic Medicine/Chemical Pathology, Guy’s & St Thomas’ Hospitals, London, UK; 4 Nuffield Department of Primary Care Health Sciences, University of Oxford, Oxford, UK; 5 The Division of Therapeutics and Molecular Medicine, University of Nottingham, Nottingham, UK

**Keywords:** Diagnosis, Hypertension

## Abstract

The 2011 NICE hypertension guideline (CG127) undertook a systematic review of the diagnostic accuracy of different blood pressure (BP) assessment methods to confirm the diagnosis of hypertension. The guideline also undertook a cost–utility analysis exploring the cost-effectiveness of the monitoring methods. A new systematic review was undertaken as part of the 2019 NICE hypertension guideline update (NG136). BP monitoring methods compared included Ambulatory BP, Clinic BP and Home BP. Ambulatory BP was the reference standard. The economic model from the 2011 guideline was updated with this new accuracy data. Home BP was more sensitive and specific than Clinic BP. Specificity improved more than sensitivity since the 2011 review. A higher specificity translates into fewer people requiring unnecessary treatment. A key interest was to compare Home BP and Ambulatory BP, and whether any improvement in Home BP accuracy would change the model results. Ambulatory BP remained the most cost-effective option in all age and sex subgroups. In all subgroups, Ambulatory BP was associated with lower costs than Clinic BP and Home BP. In all except one subgroup (females aged 40), Ambulatory BP was dominant. However, Ambulatory BP remained the most cost-effective option in 40-year-old females as the incremental cost-effectiveness ratio for Home BP versus Ambulatory BP was above the NICE £20,000 threshold. The new systematic review showed that the accuracy of both Clinic BP and Home BP has increased. However, Ambulatory BP remains the most cost-effective option to confirm a diagnosis of hypertension in all subgroups evaluated.

## Introduction

The 2011 National Institute of Health and Care Excellence (NICE) hypertension in adults guideline (CG127) [1] undertook a systematic review and economic analysis, to look at the diagnostic accuracy and cost-effectiveness of different blood pressure (BP) monitoring methods for confirming a diagnosis of hypertension. Methods compared were Ambulatory BP monitoring, Clinic BP monitoring and Home BP monitoring.

The data used for the diagnostic accuracy of the comparisons in the 2011 guideline economic analysis were based on a sensitivity analysis from the single systematic review that informed the 2011 guideline clinical review [2]. The economic analysis found that Ambulatory BP was the most cost-effective method of diagnosing hypertension [3].

As part of updating the Hypertension in adults guideline (NG136) in 2019 [4], a new systematic review on the diagnostic accuracy of BP monitoring methods for confirming a diagnosis of hypertension was performed. This new data was added to the 2011 guideline economic model to assess whether the updated accuracy data would affect the cost-effectiveness of different BP monitoring methods. All other model inputs were left unchanged.

This manuscript details both the results of the systematic review and results of the update to the economic model.

## Methods

### Diagnostic accuracy systematic review

The 2011 NICE hypertension guideline recommendations were based on a systematic review that assessed the diagnostic accuracy of different BP measurements [2]. The systematic review also undertook a sensitivity analysis whereby the sensitivity and specificity of Clinic BP (compared with Ambulatory BP) was based on a meta-analysis that excluded studies with low-mean BP. In other words, only studies with populations that had mean BPs close to or above the diagnostic threshold were included as this was more likely to represent a typical general practice screening population.

The 2019 guideline update [4, 5] conducted a new diagnostic accuracy review with the a priori inclusion and exclusion criteria specified in Table 1. There were a number of key differences between this review and that published in 2011, all of which aimed to improve the applicability of results to UK routine clinical practice. Firstly, we considered only studies published post-2000, as those before this date commonly used mercury sphygmomanometers; a method no longer used in routine clinical practice. Secondly, individuals with chronic kidney disease, established cardiovascular disease and type-2 diabetes were excluded for this diagnostic accuracy review, as they were excluded from the 2019 guideline more broadly. Thirdly, the 2011 review included studies performed in individuals who had a diagnosis of hypertension and, in some cases, were on antihypertensive treatment. These studies were also excluded as the accuracy of devices in the ‘suspected hypertension’ population might be different across a broader BP range or for those already on antihypertensive treatment. A total of five studies included in the 2011 review were therefore excluded [6–10].

**Table 1:** Differences in methods between the 2011 and 2019 NICE guideline systematic reviews.

	2011 Review [1]	2011 Review (sensitivity analysis) [1]	2019 Review [4]
Population	Adults	Adults (excluding studies with low-mean BP, i.e., excluding normotensive sample)	Adults with suspected hypertension
Target condition	Hypertension	Hypertension	Hypertension
Index tests	• Home BP • Clinic BP	• Home BP • Clinic BP	• Home BP without telemonitoring • Home BP with telemonitoring • Clinic BP • Pharmacy monitoring
Reference standard	Ambulatory BP (threshold 135/85 mmHg)	Ambulatory BP (threshold 135/85 mmHg)	Ambulatory BP (threshold 135/85 mmHg)
Outcomes	• Sensitivity • Specificity • Raw data to calculate 2 × 2 data	• Sensitivity • Specificity • Raw data to calculate 2 × 2 data	• Sensitivity • Specificity • Raw data to calculate 2 × 2 data • Studies where 2 × 2 data couldn’t be derived were included but not meta-analysed
Search strategy (data limits)	1950 onwards	1950 onwards	2000 onwards
Other exclusion criteria	• Studies where 2 × 2 data couldn’t be derived • Home with telemonitoring	• Studies where 2 × 2 data couldn’t be derived • Home with telemonitoring	– People with: • Established (already diagnosed) hypertension • Type-2 diabetes • Established cardiovascular disease • Chronic kidney disease

Full details of the systematic review, including full inclusion criteria, search strategies and data synthesis, can be found in the respective NICE guidelines [1, 4].

### Cost-effectiveness analysis update

The data used for the diagnostic accuracy of the comparisons in the 2011 guideline economic analysis were based on a sensitivity analysis from the single systematic review that informed the 2011 guideline clinical review (see Table 2) as the trials in the sensitivity analysis better reflected a suspected hypertensive population. The 2019 review identified new diagnostic accuracy data for Clinic BP and Home BP, which could change the conclusions of the previous modelling. The NICE guideline committee was interested in the comparison of Home BP and Ambulatory BP and whether this improvement in the accuracy of Home BP would change the model results. As a result, a new analysis was added to the 2011 guideline model as a minor update, in which the only input that was changed was the inclusion of the new accuracy data. All other inputs apart from the diagnostic accuracy data remained the same, and data on the methods of the previous model can be found in Appendix J of the 2011 guideline [1].

**Table 2:** Differences in evidence found between the 2011 and 2019 NICE guideline systematic reviews.

	2011 Review [1]	2011 Review (sensitivity analysis) [1]	2019 Review [4]
Total included studies	20	3	13
Studies included in Home BP meta-analysis (at threshold of 135/85 mmHg)^a^	3 studies [6, 12, 13] *N* = 561	Sensitivity analysis not conducted (mean blood pressure of all 3 studies above normotensive threshold)	4 studies [12, 14–16] *N* = 963
Studies included in Clinic BP meta-analysis (at threshold of 140/90 mmHg)^a^	7 studies [7–10, 13, 17, 18] *N* = 3693	3 studies [7, 13, 17] *N* = 809	3 studies [17, 19, 20] *N* = 1250

^a^While both reviews searched for evidence at various diagnostic thresholds, most evidence was available for the internationally accepted thresholds of 135/85 mmHg for Home BP and 140/90 mmHg Clinic BP; this data was thus most appropriate for meta-analysis.

A diagnostic meta-analysis was undertaken in WinBUGS software (version 14, University of Cambridge) for the Clinic BP and Home BP data separately. In the WinBUGS software, 60,000 paired estimates that form the joint posterior distribution for sensitivity and specificity were generated and extracted from the WinBUGS output of the diagnostic meta-analysis. In the probabilistic sensitivity analysis, a pair of sensitivity and specificity were sampled at random, thus preserving the inverse correlation. Five thousand simulations were run in the probabilistic analysis.

## Results

### Diagnostic accuracy systematic review

The 2011 review included 20 studies investigating the accuracy of different BP devices at various diagnostic thresholds compared with the Ambulatory BP reference standard. Meta-analysis was conducted with data from ten studies for the most commonly reported diagnostic thresholds (135/85 mmHg for Home BP, and 140/90 mmHg for Clinic BP, compared with a reference standard threshold of 135/85 mmHg for Ambulatory BP) [1].

The 2019 review identified 13 studies that could be included. Meta-analysis was conducted with data from ten studies from the most commonly reported diagnostic thresholds as above. The review stratified Home BP that took place with and without telemonitoring, and also stratified evidence for Clinic BP and Home BP based on the diagnostic threshold used. This left a total of four studies that were pooled for Home BP (without telemonitoring) versus Ambulatory BP, and three studies that were pooled for Clinic BP versus Ambulatory BP. A summary of the results of each review is summarised in Table 2.

The sensitivities and specificities of the measures based on the 2011 guideline (main analysis and sensitivity analysis), and the 2019 guideline, are summarised in Table 3 for comparison.

**Table 3:** Comparison of diagnostic accuracy data across 2011 and 2019 NICE guidelines systematic reviews.

	(1) 2011 Guideline clinical review^a^		(2) 2011 Guideline clinical review (sensitivity analysis)^b^		(3) 2019 Guideline clinical review^c^	
	Data	95% Confidence interval	Data	95% Confidence interval	Data	95% Confidence interval
Sensitivity						
Clinic BP	75%	61, 84	86%	81, 89	81%	47, 95
Home BP	86%	78, 91	–	–	90%	68, 98
Specificity						
Clinic BP	75%	48, 90	46%	33, 59	76%	20, 98
Home BP	62%	48, 75	–	–	84%	53, 96

Ambulatory BP is assumed to have a sensitivity and specificity of 100% as it is the reference standard.

^a^This is based on the main meta-analysis from Hodgkinson et al. [2].

^b^This is based on the sensitivity analysis from Hodgkinson et al. [2] that excluded studies with a normotensive population. This only affected the Clinic BP data.

^c^This data is based on medians because there was a skewed distribution in the accuracy data. Pooling means is only appropriate when data is normally distributed, as reported in section 9.4.5.3 of the Cochrane handbook [11]. The mean diagnostic accuracy data was used in the systematic review included as part of the 2011 guideline.

The 2019 review showed that the specificity of Home BP increased compared with the previous model inputs by around 20%, with the sensitivity also increasing slightly. When compared with the results of the sensitivity analysis from the 2011 meta-analysis for Clinic BP (after exclusion of studies with normotensive people – see column labelled 2 in Table 3), the 2019 guideline data showed that the specificity of Clinic BP measurement also increased, but sensitivity decreased slightly.

### Cost-effectiveness analysis update

The new accuracy data and their distribution based on the WinBUGS output can be seen in Table 4. This is different to that in Table 3 because medians are used for clinical meta-analysis of diagnostic accuracy data. This is because diagnostic accuracy tends to have a skewed distribution, whereas for economic analysis the means are more appropriate.

**Table 4:** New diagnostic accuracy data for model.

Input	Data	Probability distribution
Sensitivity		
Clinic BP	78%	(95% CI: 46, 95)
Home BP	88%	(95% CI: 67, 98)
Ambulatory BP	100%	Fixed
Specificity		
Clinic BP	72%	(95% CI: 20, 98)
Home BP	81%	(95% CI: 52, 96)
Ambulatory BP	100%	Fixed

Probabilistic results are summarised in Table 5 and shown graphically in Fig. A in the Appendix. Using more up to date diagnostic accuracy data confirms that a diagnosis of hypertension with Ambulatory BP following an initial raised screening BP remained the most cost-effective option in all age and sex subgroups.

**Table 5:** New diagnostic accuracy data analysis—probabilistic results.

Subgroup	Incremental QALYs versus Clinic BP		Incremental costs versus Clinic BP		Most CE strategy	Probability CE
	Home BP	Ambulatory BP	Home BP	Ambulatory BP		
Male						
Age 40	0.007 (CI: −0.013, 0.053)	0.007 (CI: −0.009, 0.047)	−£35 (CI: −£259, £190)	−£130 (CI: −£312, £29)	Ambulatory BP	88%
Age 50	0.015 (CI: −0.018, 0.081)	0.021 (CI: −0.003, 0.080)	−£26 (CI: −£179, £121)	−£85 (CI: −£219, £14)	Ambulatory BP	92%
Age 60	0.021 (CI: −0.027, 0.102)	0.034 (CI: 0.004, 0.111)	−£24 (CI: −£142, £86)	−£62 (CI: −£165, £7)	Ambulatory BP	96%
Age 70	0.022 (CI: −0.024, 0.096)	0.037 (CI: 0.009, 0.106)	−£20 (CI: −£116, £72)	−£47 (CI: −£136, £6)	Ambulatory BP	97%
Age 75	0.019 (CI: −0.021, 0.077)	0.033 (CI: 0.009, 0.084)	−£10 (CI: −£83, £52)	−£23 (CI: −£87, £16)	Ambulatory BP	98%
Female						
Age 40	0.002 (CI: −0.008, 0.023)	0.000 (CI: −0.008, 0.017)	−£50 (CI: −£350, £256)	−£186 (CI: −£406, £31)	Ambulatory BP	91%
Age 50	0.007 (CI: −0.011, 0.043)	0.009 (CI: −0.006, 0.042)	−£27 (CI: −£216, £149)	−£96 (CI: −£253, £30)	Ambulatory BP	91%
Age 60	0.011 (CI: −0.014, 0.061)	0.017 (CI: −0.001, 0.064)	−£27 (CI: −£176, £119)	−£81 (CI: −£210, £12)	Ambulatory BP	95%
Age 70	0.013 (CI: −0.015, 0.058)	0.023 (CI: 0.006, 0.065)	−£16 (CI: −£109, £71)	−£42 (CI: −£130, £14)	Ambulatory BP	97%
Age 75	0.009 (CI: −0.011, 0.041)	0.016 (CI: 0.004, 0.044)	−£7 (CI: −£97, £71)	−£21 (CI: −£106, £36)	Ambulatory BP	97%

*CE* cost-effective at a £20,000 threshold, *CI* 95% confidence interval, *QALYs* quality-adjusted life years.

Breakdowns of clinical events and costs, a summary of the number of people initially misdiagnosed, and details of how misdiagnosis changes over time, can also be found in the Appendix along with the deterministic results.

In all subgroups, both Ambulatory BP and Home BP were cost saving compared with Clinic BP, but Ambulatory BP was associated with lower costs than both Clinic BP and Home BP. In all except one subgroup (females aged 40), Ambulatory BP was associated with higher QALYs (quality adjusted life years) than Clinic BP and Home BP. Ambulatory BP was therefore dominant (both cheaper and more effective) in all except one subgroup. However, Ambulatory BP was still the most cost-effective option in 40-year-old females because the additional benefit of Home BP did not justify the additional cost (as can be seen from the incremental cost-effectiveness ratio on the top left cost-effectiveness plane in Fig. A in the Appendix) as the cost-effectiveness ratio for Home BP compared with Ambulatory BP was above the £20,000 threshold.

In the 2011 guideline model, for some of the younger male and female groups, the incremental QALYs of both Home BP and Ambulatory BP versus Clinic BP were negative, meaning that Home BP and Ambulatory BP measurement provided fewer QALYs than Clinic BP, but they were also cheaper.

## Discussion

### Diagnostic accuracy systematic review

A new systematic review on the diagnostic accuracy of methods to diagnose hypertension was undertaken as part of the 2019 NICE guideline, and the economic model of cost-effectiveness has been updated. In this updated analysis, overall the new systematic review showed that there has been an increase in the specificity of both Clinic BP and Home BP when compared with Ambulatory BP as a reference standard. This may have occurred for a number of reasons:

1. The studies themselves may be more rigorous in their methods.
2. The 2019 systematic review may be more rigorous than the 2011 systematic review, for example by removing pre-2000 studies that may have been less accurate, and only including a population that replicates the population seen in clinical practice.
3. It is also possible that differences could have occurred by chance, as it is difficult to replicate the true ‘suspected hypertension’ population in a study and the number of participants were overall fairly small.
4. The country setting of the studies may have an influence and could possibly impact the accuracy data because different countries may have different practices in how to measure and diagnose hypertension.
5. In addition, the methods used in terms of whether mean or median accuracies are reported are different in the 2011 review (reported means) and in the 2019 review (reported medians as suggested by the Cochrane handbook because the data had a skewed distribution) [11]. Medians are likely to be higher than means because diagnostic accuracy data tend to be positively skewed (have a longer right tail towards higher values). This can be seen in the final column of Table 3, where there were also wider confidence intervals from the 2019 review than from the 2011 review.

### Cost-effectiveness analysis update

Given more modern accuracy data, the update to the model shows that Ambulatory BP remains the most cost-effective method for confirming a diagnosis of hypertension for all age and sex subgroups. It is also dominant in more subgroups than previously. Ambulatory BP remains the most cost-effective method of diagnosis because the additional benefit justifies the cost.

In the 2011 guideline model, the lower QALYs of Home BP and Ambulatory BP compared with Clinic BP in some subgroups could be explained because misdiagnosing people with a measurement method that had a lower specificity (Clinic BP) led to an unexpected advantage of some people getting treatment, and therefore cardiovascular disease risk reduction, sooner, before they became hypertensive and potentially untreated in the period before they next have a BP check-up. This effect works to counteract some of the benefits of accurately diagnosing people with hypertension with more accurate methods, such as Ambulatory BP. The effect is more prominent in younger people because they have a lower prevalence of hypertension and therefore specificity plays a greater role in the results than sensitivity (see previous model write-up in the Appendix J of the previous guideline for more detail) [1]. However, in this new analysis, as the specificity of Clinic BP has increased, this anomalous effect is less prominent, and Ambulatory BP is dominant in more subgroups than it was previously, predominantly because the new accuracy data is showing higher specificities for Clinic BP and Home BP.

The initial numbers of misdiagnoses per 1000 people with suspected hypertension are shown in Table D in the Appendix. False positives and negatives over time can be seen in Figs. B, C in the Appendix. As the specificity of Clinic BP and Home BP increases, the number of false positives falls compared with the base-case analysis of the previous model. The sensitivity of Clinic BP falls in the new data used, which leads to a slight increase in the number of false negatives (those who are normotensive but have been labelled as being hypertensive and will be subjected to treatment). The number of false positives for Ambulatory BP also decreases, and it is likely that those who fail Ambulatory BP are diagnosed with Clinic BP. As the specificity of Clinic BP has improved, this leads to fewer false positives.

## Clinical conclusions

Home BP remains the best alternative method for diagnosing hypertension where Ambulatory BP is not available, however Ambulatory BP is the most cost-effective method, and likely to lead to cost savings when implemented (compared with Clinic BP and Home BP). Savings follow from correctly identifying those people who require BP treatment, and also identifying those who should not be treated.

### Summary

#### What is known about this topic

- Ambulatory BP is the most accurate method to diagnose hypertension, and is generally used as the reference standard when investigating the accuracy of Home BP and Clinic BP.
- Previous systematic reviews have shown that Home BP and Clinic BP have lower sensitivity and specificity than Ambulatory BP in the diagnosis of hypertension.
- Despite Ambulatory BP being the most expensive method, its use is cost-effective due to its diagnostic accuracy.

#### What this study adds

- A new systematic review of diagnostic accuracy of different methods of measuring BP has been undertaken as part of updating the NICE hypertension guideline.
- Compared with the 2011 hypertension guideline, sensitivity and specificity increased for Home BP, and specificity increased for Clinic BP.
- Ambulatory BP remains the most cost-effective method of diagnosing hypertension in all age and sex subgroups.

## Supplementary information

Supplementary data tables

Figure A

Figure B

Figure C
