# Supplementary material for: Options for the diagnosis of high blood pressure in primary care: a systematic review and economic model
Source: J Hum Hypertens. 2020 May 28;35(5):455–61. doi: 10.1038/s41371-020-0357-x (PMC8134050; doi:10.1038/s41371-020-0357-x)
Supplement: Supplementary file 1 — Supplementary data tables [file 41371_2020_357_MOESM1_ESM.docx]

**Supplementary data tables:**

Table A: New diagnostic accuracy data analysis - deterministic results

| **Subgroup** | **Incremental QALYs vs Clinic BP** | | **Incremental costs vs Clinic BP** | | **Optimal strategy** |
| --- | --- | --- | --- | --- | --- |
|  | **Home BP** | **Ambulatory BP** | **Home BP** | **Ambulatory BP** |  |
| **Male** | | | | | |
| Age 40 | 0.004 | 0.003 | -£49 | -£159 | Ambulatory BP |
| Age 50 | 0.011 | 0.016 | -£34 | -£102 | Ambulatory BP |
| Age 60 | 0.018 | 0.029 | -£28 | -£72 | Ambulatory BP |
| Age 70 | 0.020 | 0.034 | -£23 | -£53 | Ambulatory BP |
| Age 75 | 0.018 | 0.031 | -£13 | -£29 | Ambulatory BP |
| **Female** | | | | | |
| Age 40 | 0.000 | -0.003 | -£67 | -£218 | Ambulatory BP |
| Age 50 | 0.005 | 0.005 | -£37 | -£117 | Ambulatory BP |
| Age 60 | 0.009 | 0.014 | -£34 | -£97 | Ambulatory BP |
| Age 70 | 0.013 | 0.022 | -£21 | -£51 | Ambulatory BP |
| Age 75 | 0.009 | 0.015 | -£11 | -£29 | Ambulatory BP |

Table B: New diagnostic accuracy data analysis results (probabilistic analysis) – clinical outcome breakdown

|  | Cardiovascular events per 1,000 | | | | | Mean per person | | |
| --- | --- | --- | --- | --- | --- | --- | --- | --- |
|  | **MI** | **UA** | **SA** | **Stroke** | **TIA** | **Life years** | **QALYs** | **Disc QALYs** |
| Male, 40 years | | | | | | | | |
| Clinic BP | 125.5 | 55.4 | 163.4 | 69.5 | 23.4 | 39.12 | 31.51 | 17.35 |
| Home BP | 125.2 | 55.3 | 163.0 | 69.4 | 23.3 | 39.14 | 31.53 | 17.35 |
| Ambulatory BP | 125.3 | 55.4 | 163.3 | 69.4 | 23.4 | 39.14 | 31.52 | 17.35 |
| Male, 50 years | | | | | | | | |
| Clinic BP | 114.1 | 52.2 | 154.6 | 71.0 | 23.2 | 30.42 | 23.87 | 14.57 |
| Home BP | 113.6 | 52.0 | 154.0 | 70.7 | 23.0 | 30.45 | 23.90 | 14.58 |
| Ambulatory BP | 113.5 | 52.0 | 153.8 | 70.6 | 23.0 | 30.46 | 23.92 | 14.59 |
| Male, 60 years | | | | | | | | |
| Clinic BP | 100.5 | 48.2 | 132.1 | 69.7 | 21.3 | 22.32 | 17.12 | 11.58 |
| Home BP | 99.9 | 48.0 | 131.1 | 69.2 | 21.0 | 22.36 | 17.16 | 11.60 |
| Ambulatory BP | 99.7 | 47.9 | 130.5 | 68.9 | 20.9 | 22.39 | 17.18 | 11.61 |
| Male, 70 years | | | | | | | | |
| Clinic BP | 82.8 | 41.1 | 99.4 | 62.7 | 16.7 | 15.27 | 11.45 | 8.53 |
| Home BP | 82.0 | 40.7 | 98.4 | 61.9 | 16.4 | 15.31 | 11.49 | 8.55 |
| Ambulatory BP | 81.5 | 40.4 | 97.8 | 61.4 | 16.2 | 15.34 | 11.51 | 8.57 |
| Male, 75 years | | | | | | | | |
| Clinic BP | 71.2 | 35.8 | 84.5 | 59.3 | 13.8 | 12.26 | 9.02 | 7.01 |
| Home BP | 70.3 | 35.4 | 83.4 | 58.5 | 13.6 | 12.29 | 9.05 | 7.02 |
| Ambulatory BP | 69.7 | 35.1 | 82.7 | 57.9 | 13.5 | 12.31 | 9.07 | 7.04 |
| Female, 40 years | | | | | | | | |
| Clinic BP | 46.0 | 27.0 | 104.9 | 66.8 | 17.4 | 41.99 | 32.97 | 17.64 |
| Home BP | 45.9 | 26.9 | 104.7 | 66.6 | 17.4 | 41.99 | 32.97 | 17.64 |
| Ambulatory BP | 46.1 | 27.0 | 105.1 | 66.8 | 17.4 | 41.98 | 32.96 | 17.64 |
| Female, 50 years | | | | | | | | |
| Clinic BP | 45.2 | 23.7 | 97.5 | 68.7 | 16.6 | 32.93 | 25.06 | 14.89 |
| Home BP | 45.1 | 23.5 | 96.9 | 68.3 | 16.5 | 32.94 | 25.08 | 14.89 |
| Ambulatory BP | 45.1 | 23.5 | 96.9 | 68.4 | 16.4 | 32.94 | 25.08 | 14.89 |
| Female, 60 years | | | | | | | | |
| Clinic BP | 41.9 | 18.0 | 76.6 | 65.7 | 14.2 | 24.32 | 18.00 | 11.90 |
| Home BP | 41.7 | 17.8 | 75.8 | 65.1 | 14.0 | 24.34 | 18.02 | 11.91 |
| Ambulatory BP | 41.6 | 17.7 | 75.4 | 64.9 | 14.0 | 24.35 | 18.03 | 11.91 |
| Female, 70 years | | | | | | | | |
| Clinic BP | 33.3 | 12.0 | 50.7 | 58.3 | 12.0 | 16.59 | 11.87 | 8.69 |
| Home BP | 32.8 | 11.9 | 50.0 | 57.3 | 11.8 | 16.61 | 11.89 | 8.70 |
| Ambulatory BP | 32.5 | 11.7 | 49.5 | 56.7 | 11.7 | 16.63 | 11.91 | 8.71 |
| Female, 75 years | | | | | | | | |
| Clinic BP | 27.2 | 9.1 | 39.8 | 51.5 | 10.9 | 13.28 | 9.24 | 7.05 |
| Home BP | 26.8 | 8.9 | 39.2 | 50.6 | 10.7 | 13.30 | 9.25 | 7.06 |
| Ambulatory BP | 26.6 | 8.9 | 38.8 | 50.0 | 10.6 | 13.31 | 9.26 | 7.07 |

Table C: New diagnostic accuracy data analysis results (probabilistic analysis) – cost breakdown (mean per person)

|  | Diagnosis | Treatment | NT check-ups | MI | UA | SA | Stroke | TIA | Total cost | Disc Cost |
| --- | --- | --- | --- | --- | --- | --- | --- | --- | --- | --- |
| Male, 40 years | | | | | | | | | | |
| Clinic BP | £92 | £1,845 | £40 | £1,460 | £389 | £120 | £2,191 | £59 | £6,196 | £2,694 |
| Home BP | £104 | £1,784 | £46 | £1,454 | £388 | £119 | £2,180 | £58 | £6,133 | £2,659 |
| Ambulatory BP | £190 | £1,478 | £73 | £1,454 | £388 | £119 | £2,181 | £58 | £5,941 | £2,564 |
| Male, 50 years | | | | | | | | | | |
| Clinic BP | £76 | £1,461 | £28 | £1,156 | £325 | £105 | £2,021 | £54 | £5,225 | £2,724 |
| Home BP | £83 | £1,436 | £31 | £1,148 | £323 | £104 | £2,002 | £53 | £5,181 | £2,698 |
| Ambulatory BP | £139 | £1,265 | £46 | £1,144 | £322 | £104 | £1,996 | £53 | £5,068 | £2,640 |
| Male, 60 years | | | | | | | | | | |
| Clinic BP | £66 | £1,073 | £21 | £866 | £260 | £81 | £1,701 | £44 | £4,111 | £2,536 |
| Home BP | £70 | £1,066 | £22 | £859 | £258 | £80 | £1,678 | £43 | £4,075 | £2,512 |
| Ambulatory BP | £109 | £968 | £30 | £855 | £257 | £79 | £1,665 | £43 | £4,006 | £2,474 |
| Male, 70 years | | | | | | | | | | |
| Clinic BP | £59 | £709 | £16 | £604 | £189 | £53 | £1,234 | £29 | £2,893 | £2,074 |
| Home BP | £62 | £713 | £16 | £597 | £186 | £52 | £1,212 | £28 | £2,866 | £2,054 |
| Ambulatory BP | £91 | £657 | £20 | £592 | £185 | £52 | £1,197 | £28 | £2,823 | £2,027 |
| Male, 75 years | | | | | | | | | | |
| Clinic BP | £54 | £567 | £12 | £477 | £151 | £43 | £1,044 | £22 | £2,369 | £1,816 |
| Home BP | £55 | £582 | £12 | £470 | £149 | £42 | £1,025 | £21 | £2,356 | £1,806 |
| Ambulatory BP | £79 | £554 | £14 | £465 | £147 | £42 | £1,012 | £21 | £2,333 | £1,793 |
| Female, 40 years | | | | | | | | | | |
| Clinic BP | £108 | £1,914 | £51 | £512 | £230 | £83 | £2,185 | £47 | £5,130 | £2,064 |
| Home BP | £125 | £1,813 | £62 | £511 | £229 | £83 | £2,176 | £47 | £5,044 | £2,014 |
| Ambulatory BP | £240 | £1,377 | £101 | £513 | £230 | £83 | £2,186 | £47 | £4,775 | £1,878 |
| Female, 50 years | | | | | | | | | | |
| Clinic BP | £83 | £1,581 | £33 | £472 | £180 | £72 | £2,108 | £41 | £4,571 | £2,257 |
| Home BP | £91 | £1,544 | £37 | £469 | £179 | £72 | £2,089 | £40 | £4,522 | £2,230 |
| Ambulatory BP | £157 | £1,327 | £56 | £469 | £178 | £72 | £2,088 | £40 | £4,387 | £2,161 |
| Female, 60 years | | | | | | | | | | |
| Clinic BP | £74 | £1,147 | £26 | £391 | £114 | £50 | £1,781 | £30 | £3,613 | £2,151 |
| Home BP | £79 | £1,128 | £29 | £387 | £112 | £50 | £1,755 | £29 | £3,569 | £2,124 |
| Ambulatory BP | £129 | £982 | £40 | £386 | £111 | £50 | £1,742 | £29 | £3,469 | £2,069 |
| Female, 70 years | | | | | | | | | | |
| Clinic BP | £60 | £802 | £16 | £262 | £61 | £29 | £1,275 | £21 | £2,526 | £1,770 |
| Home BP | £62 | £811 | £16 | £257 | £60 | £28 | £1,247 | £21 | £2,501 | £1,754 |
| Ambulatory BP | £90 | £758 | £20 | £254 | £59 | £28 | £1,227 | £21 | £2,457 | £1,728 |
| Female, 75 years | | | | | | | | | | |
| Clinic BP | £57 | £628 | £14 | £193 | £40 | £21 | £989 | £18 | £1,960 | £1,471 |
| Home BP | £58 | £639 | £14 | £190 | £40 | £20 | £969 | £18 | £1,947 | £1,463 |
| Ambulatory BP | £83 | £602 | £16 | £187 | £39 | £20 | £956 | £17 | £1,921 | £1,450 |

Table D: Initial misdiagnosis per 1,000 people with suspected hypertension (deterministic)

|  | False positives | False negatives | Total misdiagnosed | False positives | False negatives | Total misdiagnosed |
| --- | --- | --- | --- | --- | --- | --- |
| 40 years | Male | | | Female | | |
| Clinic BP | 190 | 71 | 260 | 236 | 35 | 272 |
| Home BP | 125 | 38 | 163 | 156 | 19 | 175 |
| Ambulatory BP | 9 | 4 | 13 | 12 | 2 | 14 |
| 50 years | Male | | |  | | |
| Clinic BP | 143 | 106 | 249 | 169 | 87 | 255 |
| Home BP | 94 | 58 | 152 | 111 | 47 | 158 |
| Ambulatory BP | 7 | 5 | 12 | 8 | 4 | 13 |
| 60 years | Male | | |  | | |
| Clinic BP | 115 | 126 | 242 | 146 | 103 | 250 |
| Home BP | 76 | 69 | 145 | 96 | 56 | 153 |
| Ambulatory BP | 6 | 6 | 12 | 7 | 5 | 12 |
| 70 years | Male | | |  | | |
| Clinic BP | 104 | 134 | 238 | 90 | 146 | 236 |
| Home BP | 69 | 73 | 142 | 59 | 79 | 138 |
| Ambulatory BP | 5 | 7 | 12 | 4 | 7 | 12 |
| 75 years | Male | | |  | | |
| Clinic BP | 86 | 147 | 233 | 91 | 144 | 235 |
| Home BP | 57 | 80 | 137 | 60 | 78 | 138 |
| Ambulatory BP | 4 | 7 | 12 | 5 | 7 | 12 |
