# Supplementary material for: Options for the diagnosis of high blood pressure in primary care: a systematic review and economic model
Source: J Hum Hypertens. 2020 May 28;35(5):455–61. doi: 10.1038/s41371-020-0357-x (PMC8134050; doi:10.1038/s41371-020-0357-x)
Supplement: Supplementary file 2 — Figure A [file 41371_2020_357_MOESM2_ESM.pdf]

Figure A: Cost effectiveness planes for all subgroups

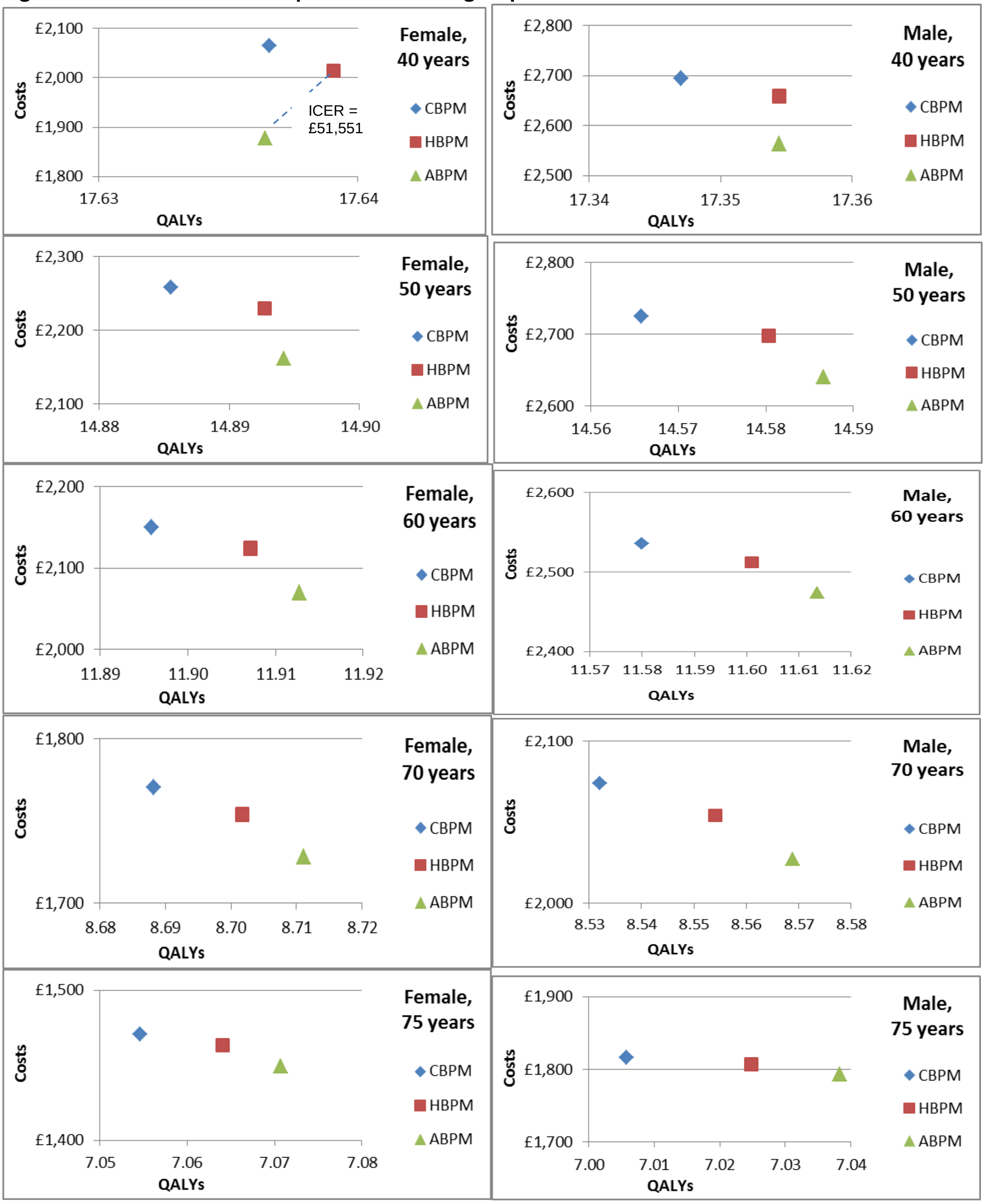

ABPM = Ambulatory BP, CBPM = Clinic BP, HBPM = Home BP, ICER=incremental cost-effectiveness ratio; QALYs=quality-adjusted life years. Where a line is shown, this represents the cost-effectiveness frontier and the ICER displayed is for HBPM compared to the lower cost intervention (ABPM) because CBPM is dominated by HBPM. In all other scenarios, ABPM dominates (higher QALYs and lower costs).
