# Supplementary material for: Options for the diagnosis of high blood pressure in primary care: a systematic review and economic model
Source: J Hum Hypertens. 2020 May 28;35(5):455–61. doi: 10.1038/s41371-020-0357-x (PMC8134050; doi:10.1038/s41371-020-0357-x)
Supplement: Supplementary file 3 — Figure B [file 41371_2020_357_MOESM3_ESM.pdf]

Figure B: Misdiagnosis over time – false positives (deterministic, male, aged 60 years)

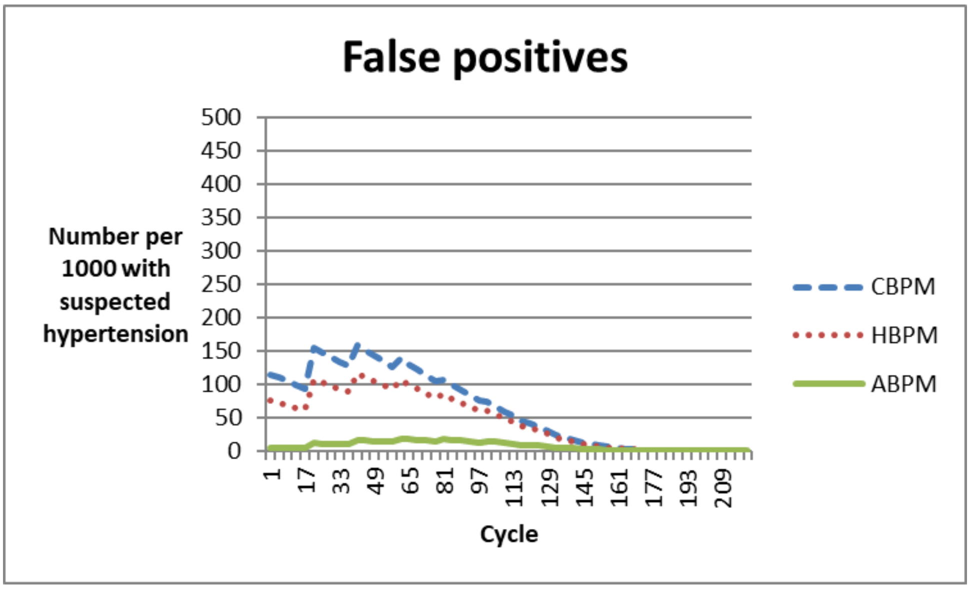

ABPM = Ambulatory BP, CBPM = Clinic BP, HBPM = Home BP.

The graph shows how the number of people in the model who have a false positive diagnosis changes over time. Overall false positives reduce over time because people develop true hypertension and become true positives. Peaks occur every 5 years when those who were diagnosed as not having hypertension have a blood pressure check-up – a certain proportion of these will have a false positive diagnosis hence the number of false positives increases. This effect diminishes over time as the number of people without a hypertension diagnosis in the model diminishes.
