# Supplementary material for: Options for the diagnosis of high blood pressure in primary care: a systematic review and economic model
Source: J Hum Hypertens. 2020 May 28;35(5):455–61. doi: 10.1038/s41371-020-0357-x (PMC8134050; doi:10.1038/s41371-020-0357-x)
Supplement: Supplementary file 4 — Figure C [file 41371_2020_357_MOESM4_ESM.pdf]

Figure C: Misdiagnosis over time – false negatives (deterministic, male, aged 60 years)

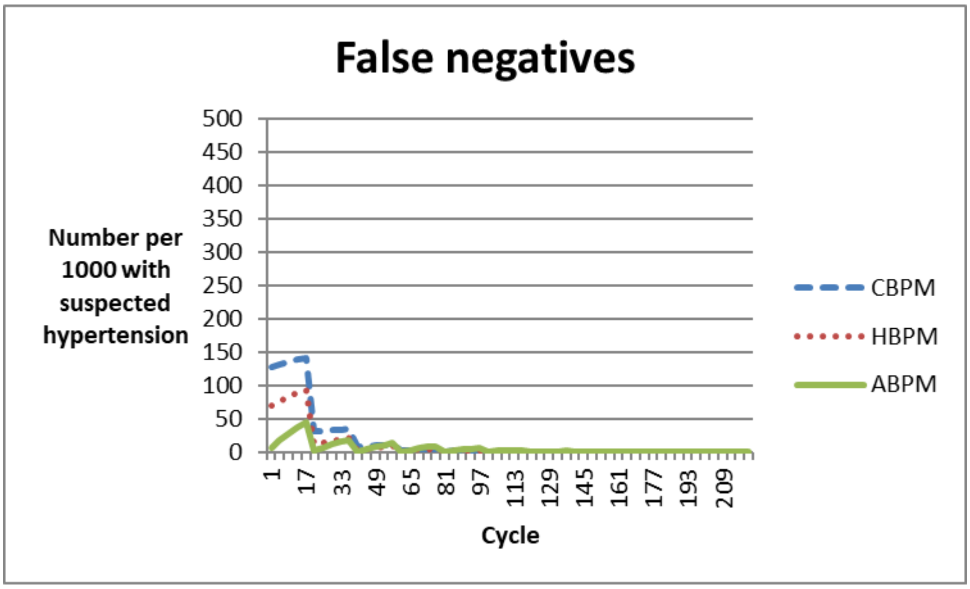

ABPM = Ambulatory BP, CBPM = Clinic BP, HBPM = Home BP.
The graph shows how the number of people in the model who have a false negative diagnosis changes over time. Between blood pressure check-ups (every 5 years) over time, the number of false negatives increases as people who were initially true negatives develop hypertension and so become false negatives. However, at each 5-year check-up a certain proportion of these are correctly identified as true positives when re-diagnosed and the graph sharply dips down.
